# Author Correction: Topical application of an irreversible small molecule inhibitor of lysyl oxidases ameliorates skin scarring and fibrosis

**DOI:** 10.1038/s41467-023-35849-x

**Published:** 2023-01-10

**Authors:** Nutan Chaudhari, Alison D. Findlay, Andrew W. Stevenson, Tristan D. Clemons, Yimin Yao, Amar Joshi, Sepidar Sayyar, Gordon Wallace, Suzanne Rea, Priyanka Toshniwal, Zhenjun Deng, Philip E. Melton, Nicole Hortin, K. Swaminathan Iyer, Wolfgang Jarolimek, Fiona M. Wood, Mark W. Fear

**Affiliations:** 1 Burn Injury Research Unit, School of Biomedical Sciences, The University of Western Australia, Crawley, Australia; 2 School of Molecular Sciences, The University of Western Australia, Crawley, Australia; 3 Drug Discovery Department, Pharmaxis Ltd, Sydney, NSW Australia; 4 School of Polymer Science and Engineering, University of Southern Mississippi, Hattiesburg, MS 39406 USA; 5 Intelligent Polymer Research Institute, Australian Research Council Centre of Excellence for Electromaterials Science, University of Wollongong, Wollongong, NSW 2500 Australia; 6 Australian National Fabrication Facility—Materials Node, Innovation Campus, University of Wollongong, Wollongong, NSW 2500 Australia; 7 Burns Service of Western Australia, WA, Department of Health, Nedlands, WA Australia; 8 Menzies Institute for Medical Research, University of Tasmania, Hobart, TAS Australia; 9 School of Pharmacy and Biomedical Sciences, Curtin University, Bentley, WA Australia; 10 School of Global Population Health, University of Western Australia, Crawley, Australia

**Keywords:** Drug development, Molecular medicine, Skin models

Correction to: *Nature Communications* 10.1038/s41467-022-33148-5, published online 22 September 2022

In this article the author name Sepidar Sayyar was incorrectly written as Sepidar Sayar. The original article has been corrected.

