# Supplementary material for: Topical application of an irreversible small molecule inhibitor of lysyl oxidases ameliorates skin scarring and fibrosis
Source: Nat Commun. 2022 Sep 22;13:5555. doi: 10.1038/s41467-022-33148-5 (PMC9500072; doi:10.1038/s41467-022-33148-5)
Supplement: Supplementary file 1 — Supplementary Information [file 41467_2022_33148_MOESM1_ESM.pdf]

Title: Topical application of an irreversible small molecule inhibitor of Lysyl Oxidases ameliorates skin scarring and fibrosis

Authors: Nutan Chaudhari[1,2], Alison D. Findlay[3], Andrew Stevenson[1], Tristan D. Clemons[2,4], Yimin Yao[3], Amar Joshi[3], Sepidar Sayar[5,6], Gordon Wallace[5,6], Suzanne Rea[7], Priyanka Toshniwal[1], Zhenjun Deng[1], Philip E. Melton[8,9,10], Nicole Hortin[1], K. Swaminathan Iyer[2], Wolfgang Jarolimek[3], Fiona M. Wood[1, 7] and Mark W. Fear[1*]

Affiliations:

[1] Burn Injury Research Unit, School of Biomedical Sciences, The University of Western Australia; Crawley, Australia.

[2] School of Molecular Sciences, The University of Western Australia; Crawley, Australia.

[3] Drug Discovery Department, Pharmaxis Ltd; Sydney, NSW, Australia.

[4] School of Polymer Science and Engineering, University of Southern Mississippi, Hattiesburg, MS, 39406, USA.

[5] Intelligent Polymer Research Institute, Australian Research Council Centre of Excellence for Electromaterials Science, University of Wollongong, Wollongong, NSW 2500, Australia;

[6] Australian National Fabrication Facility—Materials Node, Innovation Campus, University of Wollongong, Wollongong, NSW 2500, Australia

[7] Burns Service of Western Australia, WA, Department of Health; Nedlands, WA, Australia.

[8] Menzies Institute for Medical Research, University of Tasmania; Hobart, Tasmania, Australia.

[9] School of Pharmacy and Biomedical Sciences, Curtin University; Bentley, Western Australia.

[10] School of Global Population Health, University of Western Australia; Crawley.

Supplementary Materials:

Supplementary Table S1: Amine oxidase inhibition profile of PXS-4787, PXS-6302 and BAPN.

Assay	PXS-4787 IC_{50} µM (pIC_{50}±SD)	PXS-6302 IC_{50} µM (pIC_{50}±SD)	BAPN IC_{50} µM (pIC_{50}±SD)
**Human enzyme**			
Fibroblast LOX	5.19 (5.3)	4.51 (5.4 ± 0.1)	3.04 (5.5 ± 0.1)
Recombinant LOXL1	3.23 (5.5 ± 0.2)	3.42 (5.5 ± 0.1)	2.46 (5.6 ± 0.1)
Recombinant LOXL2	0.65 (6.2 ± 0.2)	0.43 (6.4 ± 0.2)	0.39 (6.4 ± 0.1)
Recombinant LOXL3	1.42 (5.9 ± 0.2)	1.54 (5.8 ± 0.1)	0.51 (6.3 ± 0.1)
Recombinant LOXL4	0.22 (6.7 ± 0.2)	0.32 (6.5 ± 0.1)	0.28 (6.6 ± 0.1)
**Dog enzyme**			
Aorta LOX	2.25 (5.7 ± 0.1)	4.74 (5.3 ± 0.1)	2.13 (5.7 ± 0.1)
Recombinant LOXL2	0.62 (6.2 ± 0.1)	0.34 (6.5 ± 0.1)	0.48 (6.3 ± 0.1)
**Rat enzyme**			
Fibroblast LOX	4.57 (5.3)	8.57 (5.1 ± 0.2)	2.12 (5.7 ± 0.1)
Recombinant LOXL2	0.59 (6.2 ± 0.1)	0.43 (6.4 ± 0.1)	0.65 (6.2 ± 0.2)
**Human amine oxidases**			
Recombinant SSAO	>100 (<4)	>30 (<4.5)	>100 (<4)
Recombinant DAO	>100 (<4)	>30 (<4.5)	>30 (<4.5)
Recombinant MAO-A	>30 (<4.5)	>30 (<4.5)	>30 (<4.5)
Recombinant MAO-B	>100 (<4)	>30 (<4.5)	>100 (<4)

Supplementary Table S2: Permeability profile of PXS-6302.

**Assay**	**PXS-6302**
LogD	1.0
$CaCo_2$ permeability	High, Papp (10^{-6} cm/s) = 34, efflux ratio 0.8
PAMPA permeability	High, GIT PAMPA at pH 7.4 Average of P(10^{-6}cm/s) = 168

Supplementary Table S3. Differentially expressed genes after PXS-4787 treatment of fibroblasts or keratinocytes *in vitro*.

ID	Log2 fold change	Adjusted p-value	Gene abbreviation	Gene name
ENSG00000167874	2.374311	0.003546	TMEM88	Transmembrane protein 88
ENSG00000069482	2.786991	0.00359	GAL	Galanin and GMAP prepropeptide
ENSG00000160223	1.738326	0.004661	ICOSLG	Inducible T cell costimulator ligand
ENSG00000180139	-3.26795	0.005156	ACTA2-AS1	ACTA2 antisense RNA 1
ENSG00000136842	*4.798825*	*0.000379*	*TMOD1*	*Tropomodulin 1*
ENSG00000231683	*3.918418*	*0.000788*	*LOC101927136*	*Uncharacterized LOC101927136*

Genes identified as being differentially expressed in fibroblasts (red) and keratinocytes (black italic) after 24 hrs of treatment with PXS-4787.

Supp Table S4 Mass Spectrometry lower limits of detection for collagen cross-linking analysis

MS Lower limit of quantification values (LLOQ)				
In vitro study PXS-4787 (Figure 4)				
HYP (pmol/10 μL)	DHLNL (pmol/10 μL)	HLNL (pmol/10 μL)	PYD (pmol/10 μL)	DPD (pmol/10 μL)
163.000	0.205	0.082	0.082	0.044
Bleomycin fibrosis model PXS-6302 (Figure 6)				
409.600	0.033	0.033	0.013	0.044
Murine excisional injury PXS-6302 (Figure 7)				
409.600	0.512	0.082	0.082	0.044
Murine excisional injury PXS-4787 (Supp. Figure S7)				
1024	0.082	0.205	0.082	0.044

Supp Table S5 Modified skin scoring scale

Modified Skin Score	Dermis and epidermis	Cell infiltrates
0	Skin appears normal	0-2 granulocyte infiltrates over 20× field
1	2x thickness compared to baseline	3-5 granulocyte infiltrates over 20× field
2	3x and greater thickness compared to baseline	6+ granulocyte infiltrates over 20× field

Supp Table S6 IHC scoring scale

IHC Score	Staining
1	Baseline
2	Mild
3	Moderate
4	Severe

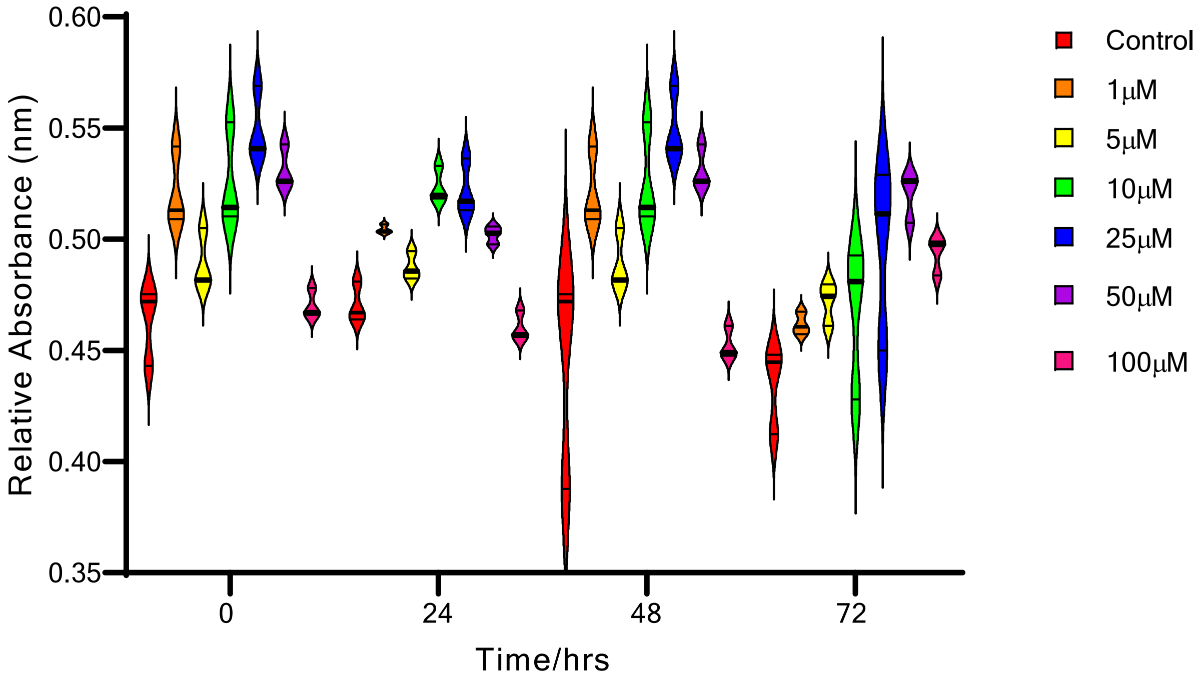

Supplementary Figure S1 Cell viability with increasing dose of PXS-4787.

In vitro cell cytotoxicity profile of PXS-4787 in dermal fibroblasts at 24, 48 and 72 hrs post treatment measured by MTS assay. Subsequent statistical analysis was performed using One-way ANOVA followed by Tukey's method for comparison. No significant difference in viability was observed.

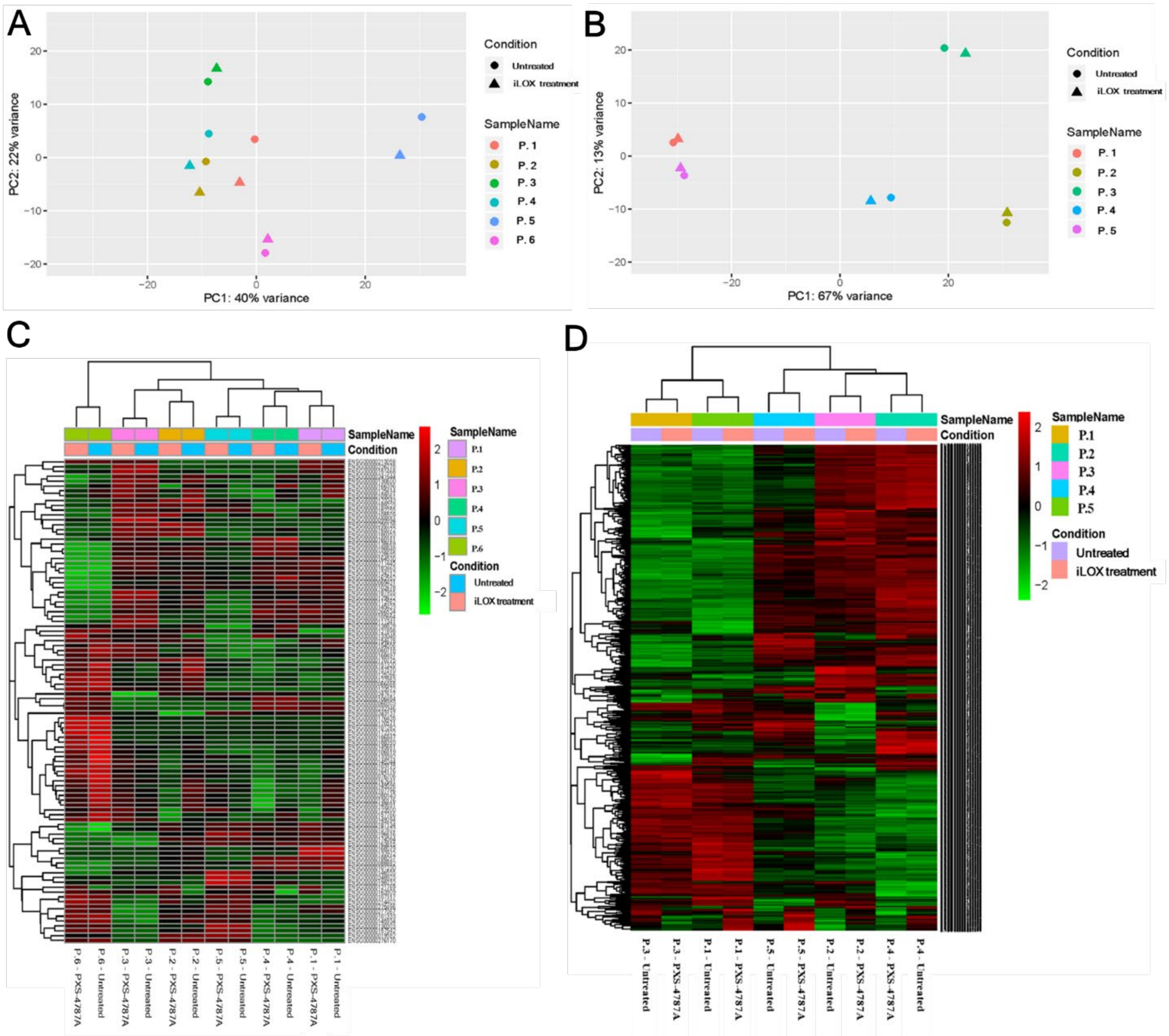

Supplementary Figure S2 Effect of PXS-4787 treatment on transcriptome of primary human dermal fibroblasts and keratinocytes.

(**A and B**) principle component analysis (PCA) score plot showing the relationship of the samples in regards to the first two principle components of the dataset was generated in primary human dermal fibroblasts and keratinocytes respectively. (**C and D**) Heatmap of data showing most variable genes in primary human dermal fibroblasts and keratinocytes respectively. Patient samples (P) were paired (n=6 in fibroblasts and n=5 in keratinocytes).

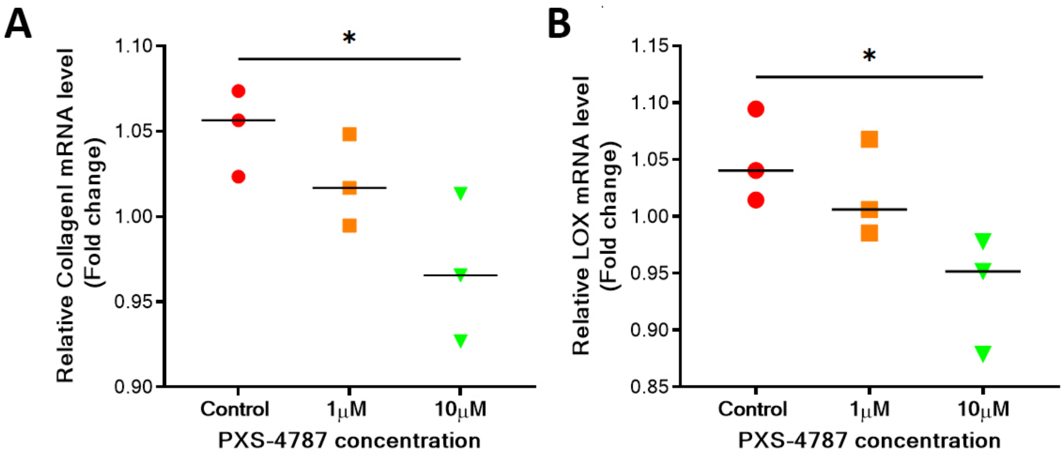

Supplementary Figure S3 qPCR for COL1A1 and LOX genes after treatment with PXS-4787.

COL1A1 and LOX mRNA levels in human dermal fibroblasts after 48 hrs treatment with PXS-4787 under scar-in-a-jar conditions. (a) Relative COL1A1 expression ($p=0.050$) (b) Relative LOX mRNA expression ($p=0.0491$). Subsequent statistical analysis was performed using One-way ANOVA followed by Tukey's method for comparison ($*p<0.05$)

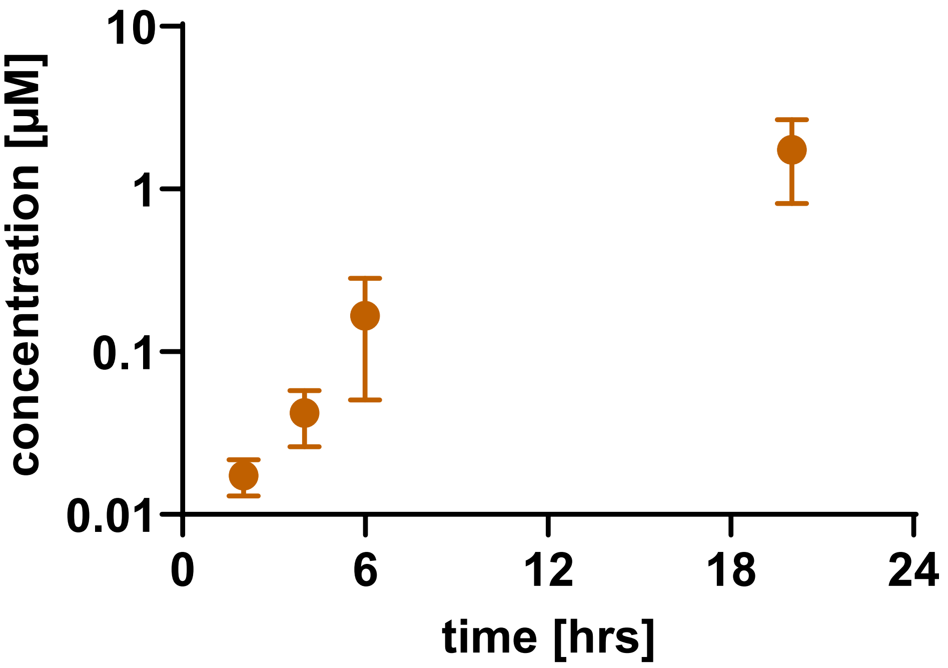

Supplementary Figure S4: PXS-6302 concentration increases over time in receiving compartment in Franz Cell experiment using human skin demonstrating skin permeability. Time dependent increase in receiving compartment from 3 separate experiments using full-thickness human skin from different adult donors and body sites. Saturating doses of cream were applied to the epidermis in the donor chamber of the apparatus. Diffusion of PXS-6302 was measured using the concentration of PXS-6302 observed over time in the receptor chamber below the dermal layer of the skin. Data is presented as mean +/- SEM.

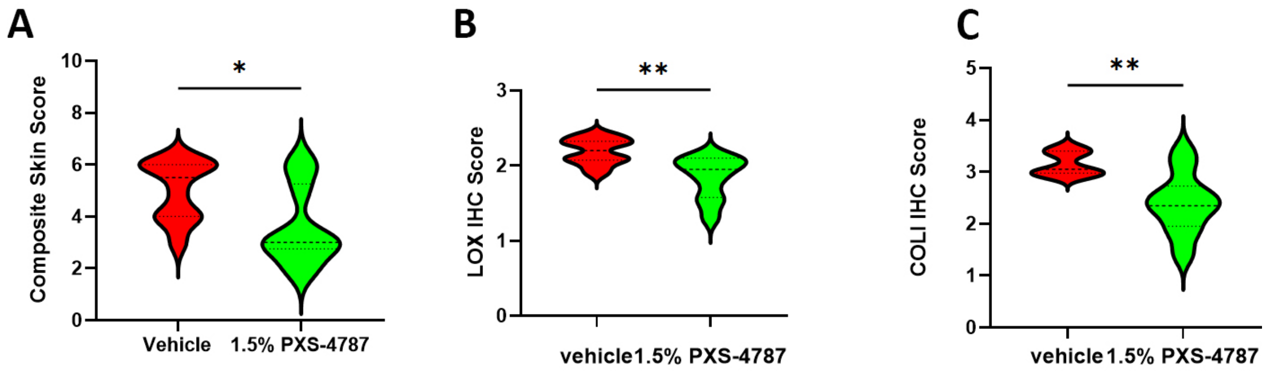

Supplementary Figure S5 Topical PXS-4787 treatment reduces bleomycin-induced skin fibrosis

(A, B and C) Improved skin fibrosis after topical application of PXS-4787 in bleomycin- induced fibrosis model in mice (n=5). **(A)** Reduced composite skin fibrosis score after 1.5% PXS-4787 treatment group (p=0.0284). (**B)** Decreased subjective LOX score (p=0.0049) and **(C)** decreased subjective COL1 score (p=0.0041) in the 1.5% PXS-4787 topical treatment group (n=10 animals per group). Statistical analysis was performed with two-tailed Mann-Whitney test, *p<0.05, **p <0.01.

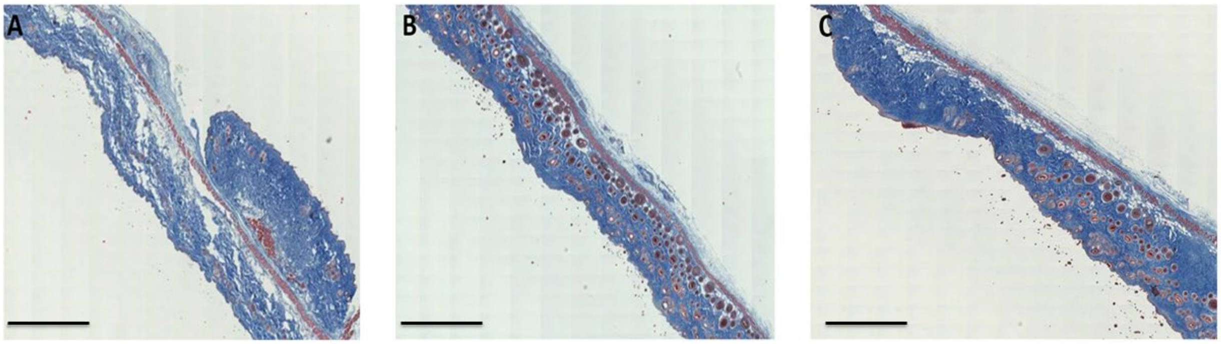

Supplementary Figure S6 Topical PXS-6302 treatment reduces bleomycin-induced skin fibrosis

Masson's trichrome staining was used to assess composite skin scores by blinded assessors according to Supp. Table S5**.** Reduced skin fibrosis after topical application of PXS-6302 in bleomycin-induced fibrosis model in mice was observed (n=10 per group) **(A)** Naïve mouse skin (**B)** Bleomycin treated with vehicle (**C)** Bleomycin treated with PXS-6302. Scale bar is 1mm.

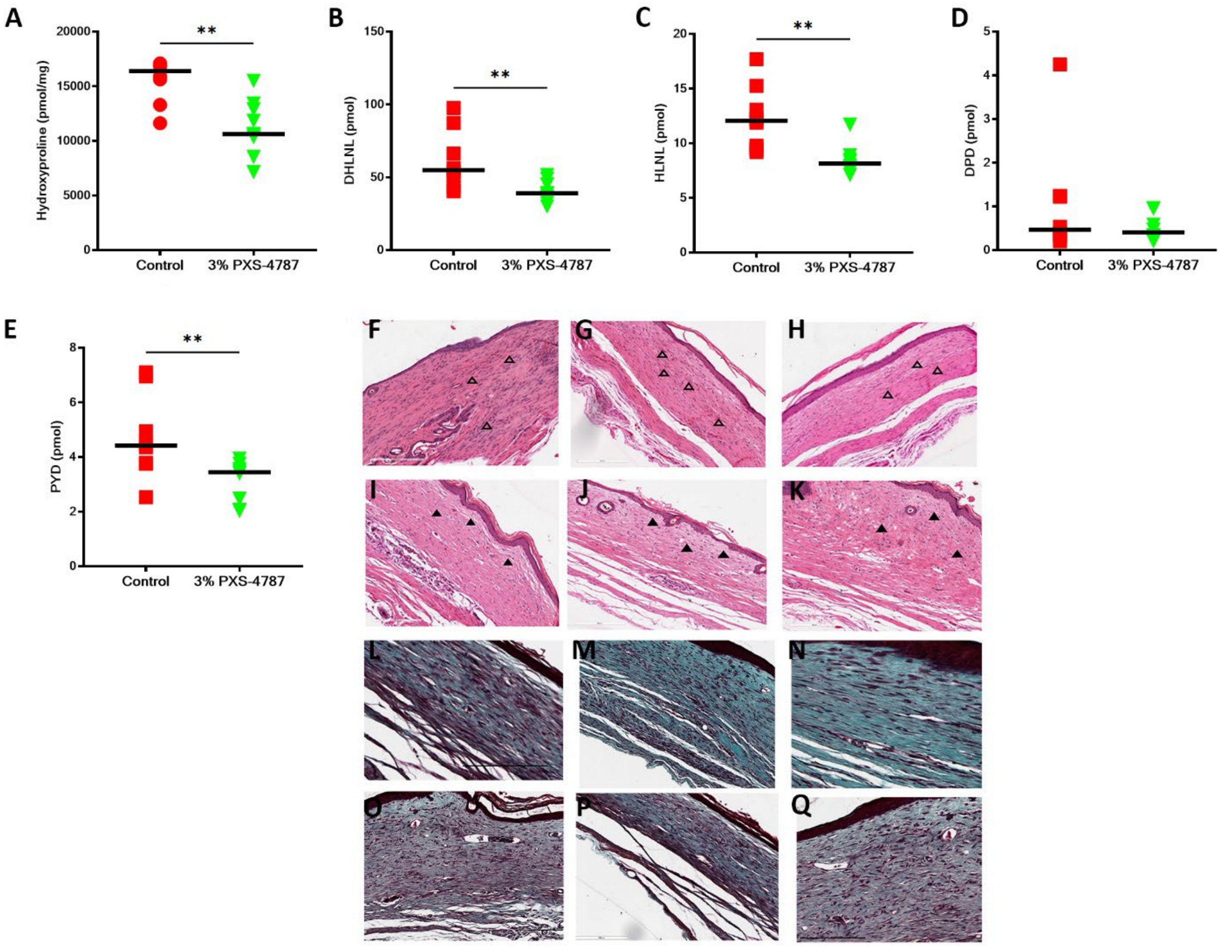

Supplementary Figure S7. Topical PXS-4787 treatment improves scar formation in a full-thickness excision injury model in mice

(A-E) Reduced collagen cross-links formation in mouse scar after topical treatment of 3% PXS-4787 cream for 28 days in excision injury mouse model (n=8). **(A)** Hydroxyproline (p=0.025), **(B)** DHLNL (p=0.0079), **(C)** HLNL (p=0.0011), **(D)** DPD, **(E)** PYD (p=0.0070). **(F-K)** Representative H&E staining images of mouse skin showing changes in the collagen structure following PXS-4787 treatment. **(F-H)** images of control scar with open arrows highlighting thick, aligned collagen bundles. **(I-K)** PXS-4787-treated scar with filled arrows highlighting loosely packed collagen. **(L-Q)** Representative Masson's Trichrome staining images of mouse skin

showing changes in the collagen structure following PXS-4787 treatment. **(L-N)** images of control scar with dense and parallel aligned collagen bundles. **(O-Q)** PXS-4787-treated scar with less dense and less aligned collagen structure. Scale bar is 200 μm (n=8-9 animals per group). Statistical analysis was performed with two-tailed Mann-Whitney test, $^{*}p<0.05$, $^{**}p<0.01$

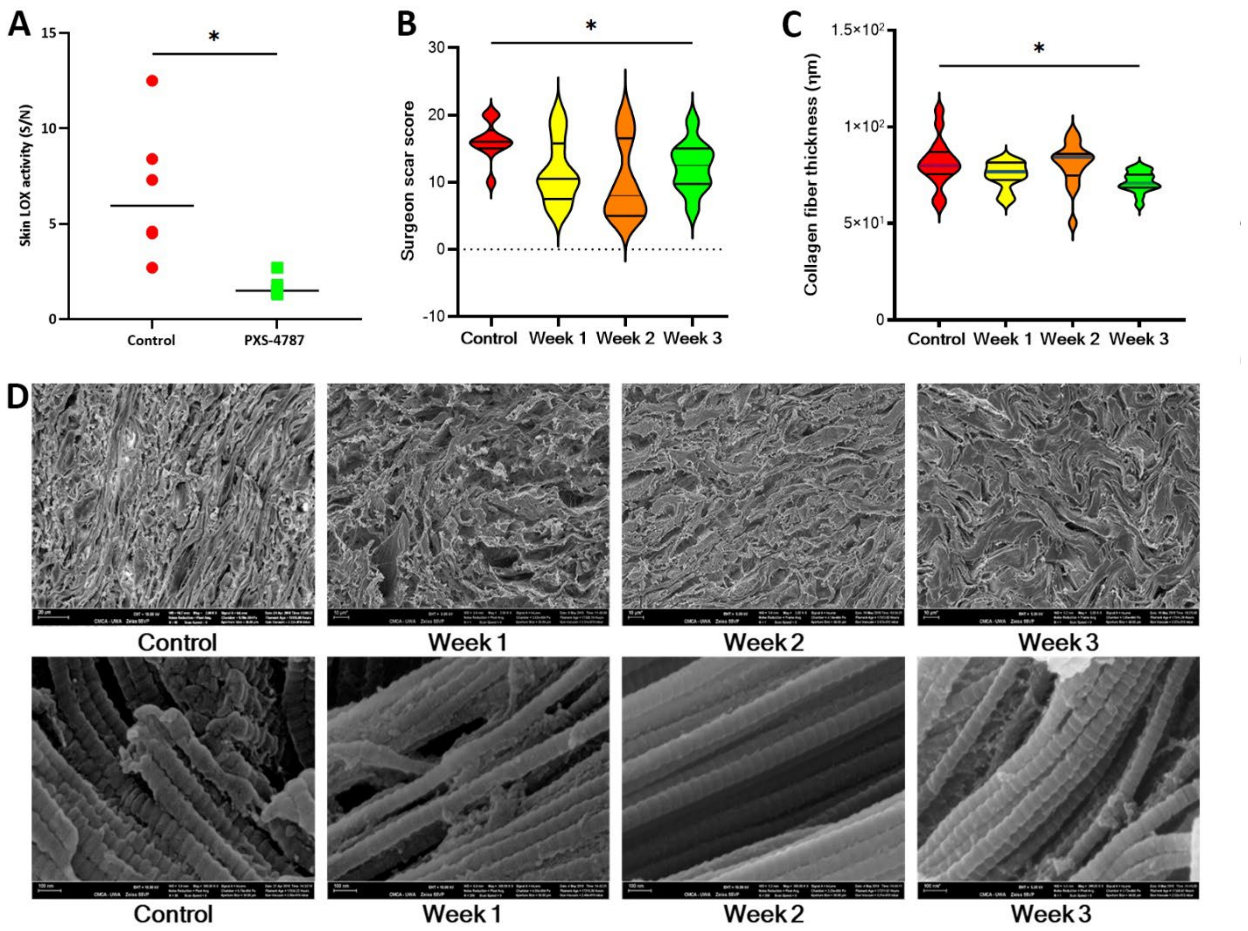

Supplementary Figure S8 Topical PXS-4787 treatment inhibits LOX enzymes and improves scar appearance in a porcine model of injury.

(A) Lox enzyme activity significantly inhibited in PXS-4787 treated scars 24 hours after final application of treatment (p=0.0043). (**B)** Scars ranked in matched sets of four (for one of each treatment group on same animal and body site, 0 best scar to 3 worst scar) by plastic surgeons blinded to treatment shows significant improvements in scar appearance with treatment in the group that commenced treatment at 3 weeks post-injury (p=0.0240). Treatment was commenced at 1, 2 or 3 weeks after injury, and all scars analysed at 12 weeks post-injury **(C, D)** Collagen fiber thickness measured using SEM shows significant reduction in the treatment group with treatment that commenced at 3 weeks post-injury ((p=0.0287) n=10 excisional wounds per treatment group,

LOX activity n=6 per group). Statistical analysis was performed with two-tailed Mann-Whitney test (A) or Repeated measures ANOVA with Tukey's methods for multiple comparisons (B) or one-way ANOVA with Tukey's method for multiple comparisons (C), *$p<0.05$

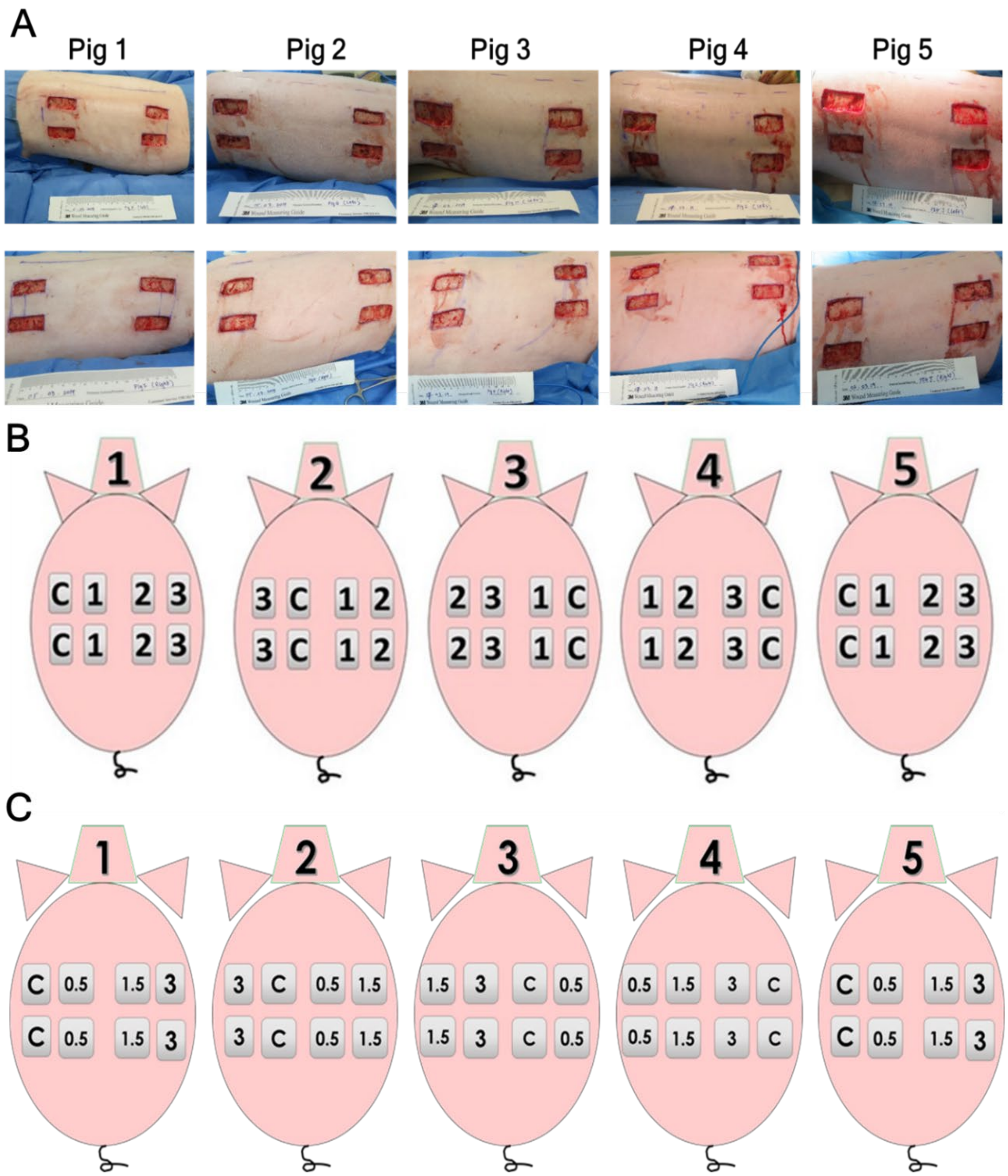

Supplementary Figure S9 Schematic representation of full-thickness excision injury model in pigs.

(A) Representative photos of excision injury in pigs. **(B)** Schematic representation of treatment regimen in porcine excision injury model Where C = control treatment, 1 = 3%

PXS-4787 treatment started at week 1 post excision, 2 = 3% PXS-4787 treatment started at week 2 post excision and 3 = 3% PXS-4787 treatment started at week 3 post excision injury. **(C)** Schematic representation of treatment regimen in porcine excision injury model where C = control, 0.5 = 0.5% PXS-6302 treatment, 1.5 = 1.5% PXS-6302 treatment and 3 = 3% PXS-6302 treatment.

A

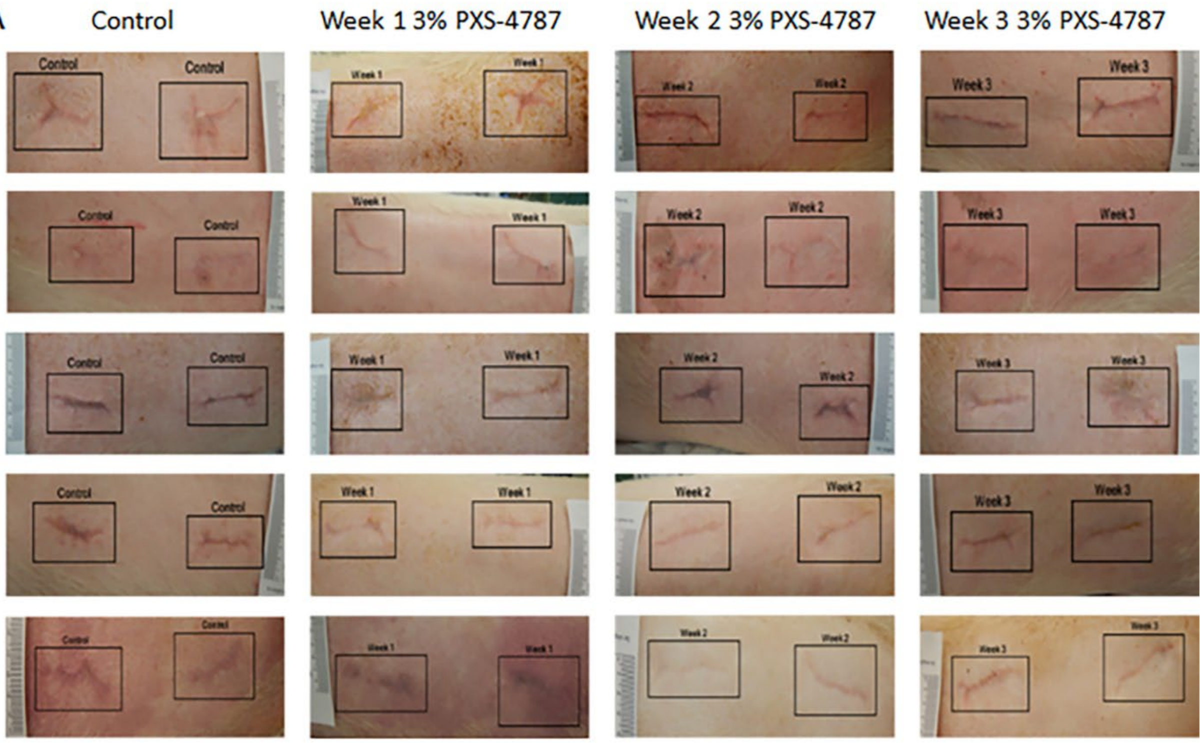
Control
Week 1 3% PXS-4787
Week 2 3% PXS-4787
Week 3 3% PXS-4787

B

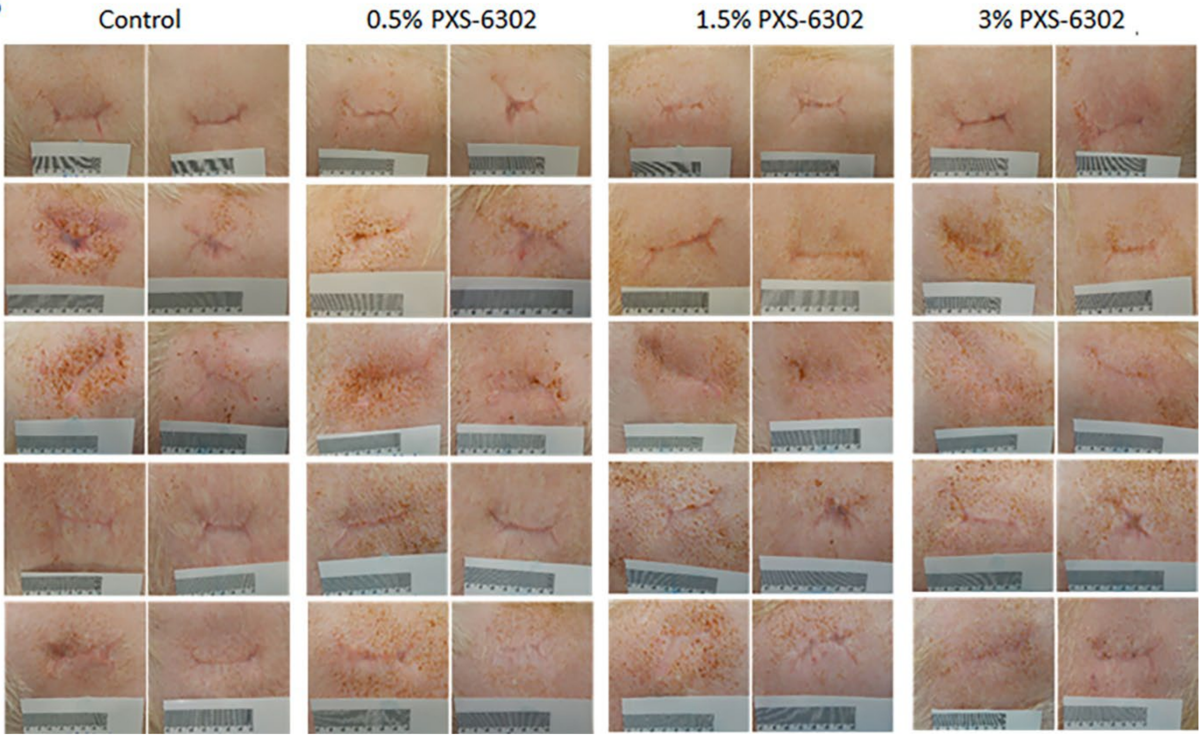
Control
0.5% PXS-6302
1.5% PXS-6302
3% PXS-6302

Supplementary Figure S10 Photos of scars at time of euthanasia

(A) Photos of scars treated with vehicle or 3% PXS-4787. Treatment was commenced at one week, two weeks or three weeks post injury and continued once daily in all groups until 12-weeks post injury **(B)** Photos of scars treated with vehicle or 0.5%, 1.5% or 3% PXS-6302 from time of re-epithelialisation until 12-weeks post injury
