# Supplementary material for: Topical application of an irreversible small molecule inhibitor of lysyl oxidases ameliorates skin scarring and fibrosis
Source: Nat Commun. 2022 Sep 22;13:5555. doi: 10.1038/s41467-022-33148-5 (PMC9500072; doi:10.1038/s41467-022-33148-5)
Supplement: Supplementary file 3 — Description of Additional Supplementary Files [file 41467_2022_33148_MOESM3_ESM.pdf]

Description of Additional Supplementary Files

Supplementary Data 1: LeadProfilingScreen (Eurofins Cerep Panlabs) for PXS-4787.

Supplementary Data 2: SafetyScreen (Eurofins Cerep Panlabs) for PXS-6302.
